# Dataset for case studies of hydropower unit commitment

**DOI:** 10.1016/j.dib.2018.03.015

**Published:** 2018-03-08

**Authors:** Jinwen Wang, Hao Zheng, Yan Li, Teng Ma

**Affiliations:** a Joint International Water Security Research Center, Huazhong University of Science and Technology, Wuhan, Hubei 430074, China; b School of Hydropower and Information Engineering, Huazhong University of Science and Technology, Wuhan, Hubei 430074, China

## Abstract

This paper presents the data all needed for nine case studies of hydropower unit commitment, which determines the optimal operating zones and generating discharges of units after the quarter-hourly releases and water heads are derived by the operation of cascaded hydropower reservoirs. The power output function and feasible operating zones of units are provided, and optimization solvers are used to acquire the results in detail for the case studies, including the quarter-hourly generating discharges, power generations, as well as operating zones of individual units. Performance indices, including the spillage, energy production, and the low-efficiency generating rate, are summarized for all case studies and can be readily used for comparison between algorithms in future.

**Specifications Table**

**Table:** 

Subject area	*Energy Management*
More specific subject area	*Hydropower unit commitment*
Type of data	*Tables and figures*
How data was acquired	*Unit power output characteristics are estimated based on observed data; Releases and water heads are provided in Ref**[*1*]**; and results for nine case studies are obtained with optimization solvers.*
Data format	*Filtered and analyzed*
Experimental factors	*IBM CPLEX 12.6 solver called by C++ codes, which are executed on an HP laptop [Intel(R) Core(TM)2 duo CPU T5550 @ dual 1.83 GHz]*
Experimental features	*The CPLEX 12.6 solver to derive the unit operating zones, and the Hill-Climbing method to determine the unit generating discharges and power generations.*
Data source location	*Yunnan, China*
Data accessibility	*Attached to this article as an excel file.*

**Value of the data**

• Nine case problems in different sizes are presented in detail for researchers to test, compare, and choose optimization solvers for mixed integer linear programming.
• The parameters are very useful for upcoming algorithms to test their efficiency in optimizing quarter-hourly hydropower unit commitments, which is one of the most significant optimization problems in power systems.
• The results obtained herein with a mixed integer linear programming (MILP) solver can serve as a standard benchmark for other researchers to compare their results with.
• The case studies can be easily scaled up to problems in larger size to test optimization solvers or algorithms on their capability in solving large-scale problems of hydropower unit commitment.

## 1 Data

**Fig. 1:**
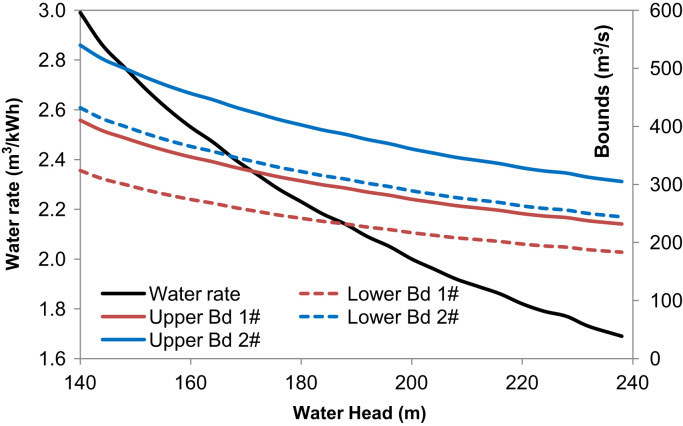
Water rate and feasible operating zones of a unit.

Table A1 in the attached excel file gives the water rates, feasible lower and upper bounds on the generating discharge, which are all functions of the water head and plotted in Fig. 1. The water rate can be used to calculate the power output given a generating flow at a certain water head.

**Fig. 2:**
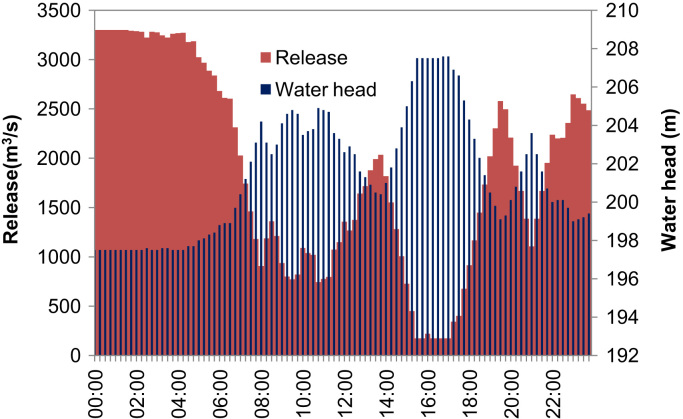
Quarter-hourly water heads and releases of the reservoir.

The quarter-hourly water heads and releases are known parameters, which are listed in Tables A2–A10 in the attached excel file for nine case studies respectively. Fig. 2 shows the quarter-hourly water heads and releases given in Table A10 for the 9^th^ case study that involves nine units.

The performance indices are also summarized in Tables A2–A10 for each case study, including the total spillage, total energy production, low-efficiency generating rate, and the computation time. The optimal quarter-hourly generating flows, power generations and operating zones of units, as well as the optimal quarter-hourly spillages and power generations of the hydropower reservoir, are also obtained and given in Tables A2–A10 for nine case studies respectively.

## 2 Experimental design, materials and methods

### 2.1 Unit power output characteristics

The lower and upper bounds on the generating flow of a unit at a certain water head in Table A1 are estimated with engineering experience. The relationship between the water head in meters and the water rate (η) in m^3^/kWh in Table A1 are estimated based on observed power outputs (P) in MW and generating discharges (Q) in m^3^/s at different water heads, mathematically expressed as:

(1) $$\eta =\frac{3.6Q}{P}$$

### 2.2 Hydrological parameters

The data of the 9th case study with 9 units involved are given in Table A10 that includes the quarter-hourly water heads ($h_{t}$) and releases ($Q_{t}$) of the reservoir, which are provided by a model presented in Ref [1] for short-term hydropower scheduling of cascaded reservoirs. All nine case studies in Tables A2–A10 use the same quarter-hourly water heads, but different quarter-hourly releases (${\hat{Q}}_{t}$) determined for any case study with *n* units available by a linear scaling:

(2) $${\hat{Q}}_{t}=\frac{n}{9}Q_{t}$$

### 2.3 Results of case studies

Tables A2–A10 present the optimal quarter-hourly generating flows ($q_{it}$) in m^3^/s, power generations ($p_{it}$) in MW and operating zones of individual units for nine case problems respectively. These results are derived by a mixed integer linear programming and the hill-climbing method, detailed in Ref [2]. The quarter-hourly spillages ($spl_{t}$) in m^3^/s and power outputs ($P_{t}$) in MW of the hydropower reservoir are given in Tables A2–A10 and calculated respectively for each case study by:

(3) $$spl_{t}={\hat{Q}}_{t}-\sum_{i=1}^{n}q_{it},$$

(4) $$P_{t}=\sum_{i=1}^{n}p_{it},$$

where i is the index of units, and *n* is the number of units involved in the case study.

### 2.4 Performance indices for each case study

The performance indices summarized in Tables A2–A10 for each case study include the total spillage (S) in m^3^, total energy production (E) in GWh, computation time, and the numbers of shutdowns (N_0_), low-efficiency zones (N_1_) and high-efficiency zones (N_2_) committed to for all time-steps and of all units involved in the case study, as well as the low-efficiency rate (λ). The computation time is calculated by recording the time at both the beginning and end of the program execution. The total spillage is calculated by:

(5) $$S=\sum_{t=1}^{96}\left(900\cdot spl_{t}\right),$$

where there are 96 quarter-hours during a day, with a quarter-hour equal to 900 s. The total energy production is determined by:

(6) $$E=\sum_{t=1}^{96}\left(\frac{1}{4}\cdot \frac{1}{1000}P_{t}\right),$$

where the length of a time-step is one fourth hour, and one thousandth is a coefficient used to convert the power output in MW to that in GW. The numbers of shutdowns (N_0_), low-efficiency zones (N_1_) and high-efficiency zones (N_2_) are determined by counting the optimal operating zones equal to 0, 1 and 2, respectively. And the low-efficiency rate (λ) is obtained by:

(7) $$\lambda =\frac{N_{1}}{N_{0}+N_{1}+N_{2}}$$
