# Supplementary figures and images for: Dataset for case studies of hydropower unit commitment
Source: Data Brief. 2018 Mar 8;18:139–43. doi: 10.1016/j.dib.2018.03.015 (PMC5996229; doi:10.1016/j.dib.2018.03.015)

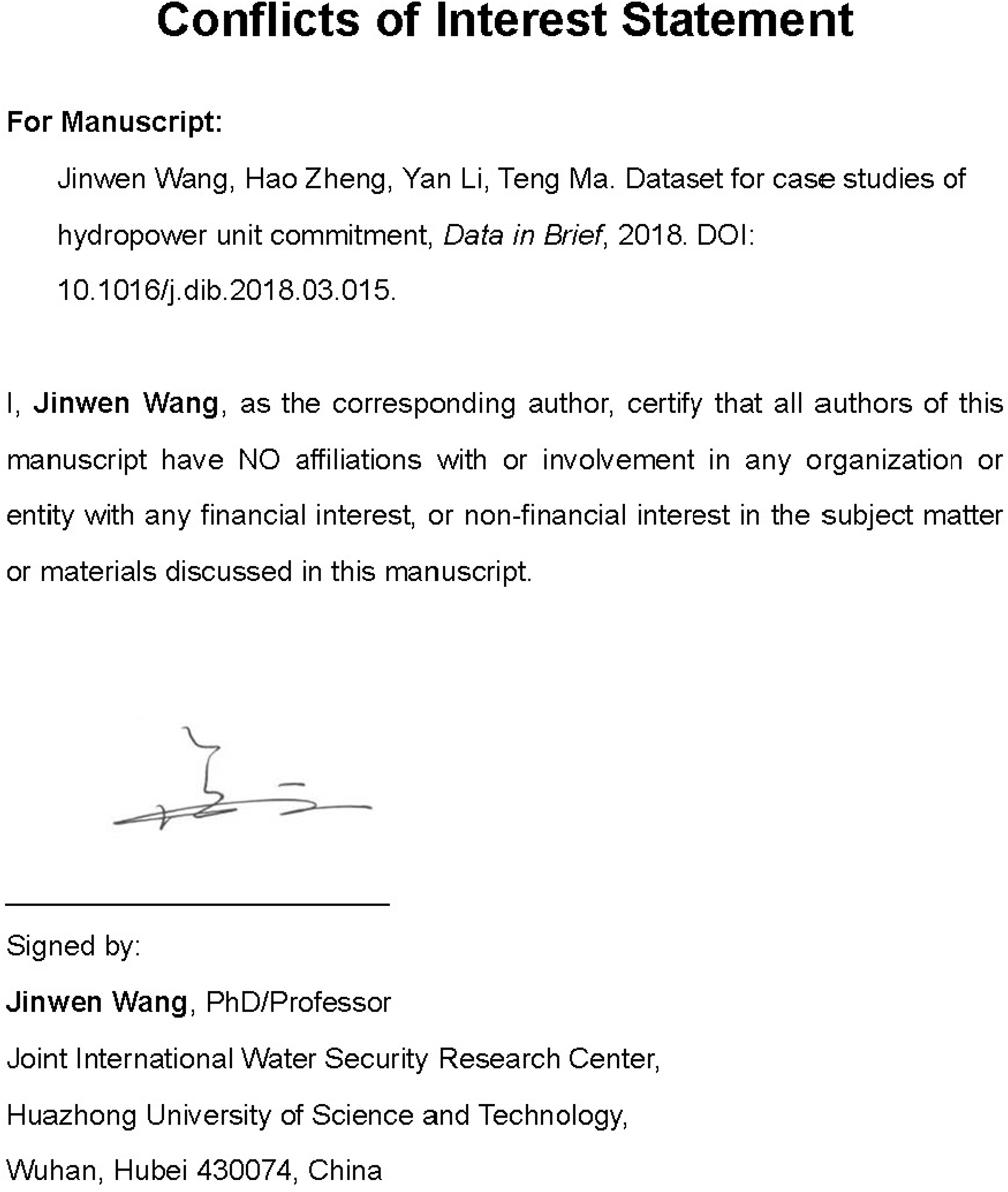

Supplement: Supplementary file 1 — Supplementary material [file mmc1.jpg]
